# Plasmonic bacteria on a nanoporous mirror via hydrodynamic trapping for rapid identification of waterborne pathogens

**DOI:** 10.1038/s41377-018-0071-4

**Published:** 2018-10-03

**Authors:** Keumrai Whang, Jong-Hwan Lee, Yonghee Shin, Wooju Lee, Young Wan Kim, Dongchoul Kim, Luke P. Lee, Taewook Kang

**Affiliations:** 1 Department of Chemical and Biomolecular Engineering, Sogang University, Seoul, 04107 Korea; 2 Berkeley Sensor and Actuator Center, Departments of Bioengineering, Electrical Engineering and Computer Science, Biophysics Graduate Program, University of California, Berkeley, Berkeley, CA 94720 USA; 3 Department of Mechanical Engineering, Sogang University, Seoul, 04107 Korea

## Abstract

A rapid, precise method for identifying waterborne pathogens is critically needed for effective disinfection and better treatment. However, conventional methods, such as culture-based counting, generally suffer from slow detection times and low sensitivities. Here, we developed a rapid detection method for tracing waterborne pathogens by an innovative optofluidic platform, a plasmonic bacteria on a nanoporous mirror, that allows effective hydrodynamic cell trapping, enrichment of pathogens, and optical signal amplifications. We designed and simulated the integrated optofluidic platform to maximize the enrichment of the bacteria and to align bacteria on the nanopores and plasmonic mirror via hydrodynamic cell trapping. Gold nanoparticles are self-assembled to form antenna arrays on the surface of bacteria, such as *Escherichia coli* and *Pseudomonas aeruginosa*, by replacing citrate with hydroxylamine hydrochloride in order to amplify the signal of the plasmonic optical array. Owing to the synergistic contributions of focused light via the nanopore geometry, self-assembled nanoplasmonic optical antennas on the surface of bacteria, and plasmonic mirror, we obtain a sensitivity of detecting *E. coli* as low as 10^2^ cells/ml via surface-enhanced Raman spectroscopy. We believe that our label-free strategy via an integrated optofluidic platform will pave the way for the rapid, precise identification of various pathogens.

## Nanoparticles: making it easier to detect waterborne pathogens

A novel method using nanotechnology to rapidly identify bacteria in water samples has been developed by scientists in the Republic of Korea and the US. Waterborne pathogens kill millions of people each year, but detection methods can be time-consuming, lack sensitivity, and only work with small samples. Taewook Kang at Sogang University, Seoul, and co-workers fabricated a gold-coated mirror filled with nano-sized holes smaller than an individual bacterium, and added especially modified gold nanoparticles to their water samples. The nanoparticles stuck onto the surface of bacteria, such as *Escherichia coli*, which in turn made the bacteria likely to cluster around the mirror’s pores. These measures greatly enhanced the interactions between the bacteria and light, so that even very low cell concentrations could be detected using spectroscopy, and different species of bacteria could be distinguished.

## Introduction

Waterborne pathogen-related diseases are global health issues, leading to > 2.2 million deaths per year (with 1.5 million of these deaths being reported for children)^1,2^. Conventional diagnostic techniques for waterborne pathogens, especially bacteria, are largely based on either selective culturing or molecular diagnosis, including immunoassays and the polymerase chain reaction. Although selective culturing has been considered the gold standard for the identification of waterborne bacteria, this technique is limited by its time-consuming processes (typically taking 1–2 days for routine identification), lack of sensitivity and specificity, and difficulties related to cultures (sometimes nonculturable)^3–6^. Compared with selective culturing, molecular diagnosis exhibits better sensitivity and a faster detection time. However, this technique requires an additional sample preparation step to enrich the bacteria, which are dispersed in a large volume of water^7^. Moreover, this process still takes several hours and needs expensive reagents and equipment^8,9^.

On the other hand, nanostructure-based optical methods have gained increasing attention because of their high sensitivity and rapid detection time^10–22^. Among them, surface-enhanced Raman spectroscopy (SERS) is particularly attractive for the ultrasensitive detection of bacteria^17–22^. Metallic nanostructures, including colloidal nanoparticles modified with specific antibodies or Raman active dyes or magnetic nanoparticles for the enrichment of bacteria, have also been demonstrated. However, the previously proposed SERS-based detection methods are generally limited to small sample volumes of a few microliters. Considering that waterborne bacteria are dispersed at very low concentrations, processing small sample volume undermines the reliability of detection. Therefore, besides high sensitivity and rapid detection time, an ideal SERS-based method for detecting waterborne bacteria should be capable of treating large sample volumes.

**Fig. 1:**
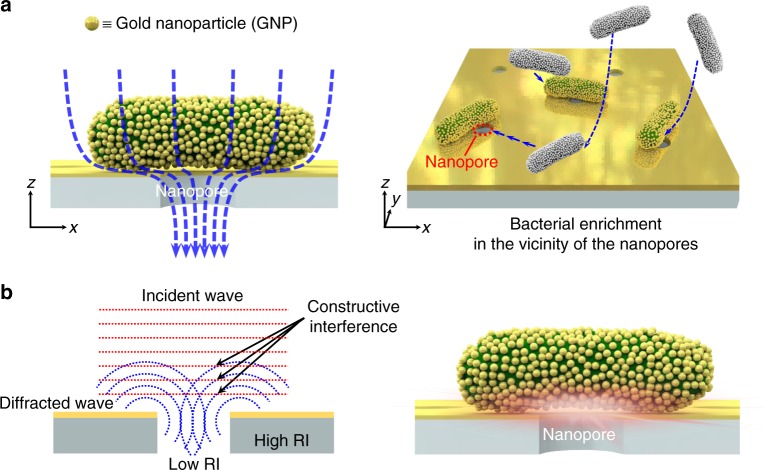
Plasmonic bacteria on a nanoporous mirror membrane **a** Schematic illustration of hydrodynamic trapping of plasmonic bacteria on nanopores. Owing to the hydrodynamic force on the bacteria surface, GNP-assembled bacteria (plasmonic bacteria) are forced to move along the flow and are located on the nanopore. **b** Schematic illustration of the constructive interference between incident light and diffracted light at the fringe of the nanopore (left). The constructive interference leads to strong near-field enhancement between the GNPs on the plasmonic bacteria as well as the GNPs and the gold mirror (right)

Here, we report plasmonic bacteria on a nanoporous mirror via hydrodynamic trapping, which allows enrichment of bacteria from large sample volumes and strong signal amplifications for the rapid identification of bacteria. Our design is schematically illustrated in Fig. 1. First, gold nanoparticles (GNPs) are spontaneously self-assembled to form antenna arrays on the surface of bacteria (plasmonic bacteria). Then, plasmonic bacteria are forced to be located on the nanopore of the membrane as a result of hydrodynamic trapping (Fig. 1a). Hydrodynamic trapping enables the enrichment of bacteria on the nanopore from large sample volumes. Once bacteria are trapped on the pore, owing to the synergistic contributions of (1) focused light by constructive interference between incident light and its diffraction via the nanopore, (2) self-assembled nanoplasmonic antennas on the surface of bacteria, and (3) plasmonic mirrors, a strong near-field enhancement between GNPs on plasmonic bacteria, as well as between GNPs on plasmonic bacteria with a gold thin mirror around the nanopore, (Fig. 1b) is expected. Sensitive label-free optical detection of bacteria from large sample volumes would therefore be possible with this design.

## Results

**Fig. 2:**
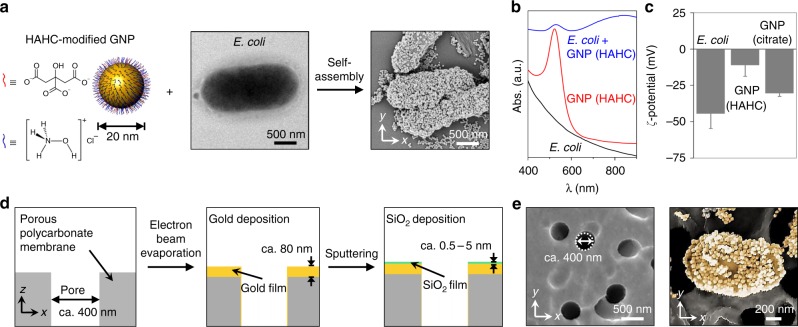
Self-assembly of gold nanoparticles on bacteria and fabrication of the nanoporous mirror **a** Self-assembly of hydroxylamine hydrochloride (HAHC)-modified gold nanoparticles (GNPs) on the surface of *E. coli*. Schematic illustration of HAHC-modified GNPs (left), the representative transmission electron microscope image of *E. coli* (middle), and scanning electron microscope (SEM) image of GNP-assembled *E. coli* (plasmonic *E. coli*, right). **b** UV-vis spectra of the *E. coli* suspension, HAHC-modified GNP solution, and mixture of HAHC-modified GNP and *E. coli*. **c** Zeta-potentials of *E. coli*, HAHC-modified GNP, and citrate-capped GNP. **d** Schematic illustration for the fabrication of the nanoporous mirror via serial deposition; electron beam evaporation for the gold thin film and sputtering for the SiO_2_ layer. **e** Representative SEM images of the resulting nanoporous mirror (left) and plasmonic bacteria on a nanoporous mirror (right)

For the self-assembly of GNPs on the bacterial surface, hydroxylamine hydrochloride (HAHC) is used to reduce the strongly negative surface charge of citrate-capped GNPs by replacing citrate ions with HAHC. Then, HAHC-modified GNPs with a diameter of 20 nm are mixed with *Escherichia coli* (*E. coli*). The representative scanning electron microscope (SEM) image in Fig. 2a, taken after mixing, shows that *E. coli* cells are densely covered by GNPs. To further verify the self-assembly of GNPs on the surface of *E. coli*, UV-vis spectra were measured before and after the mixing. Figure 2b shows the absorbance spectra of an *E. coli* solution, HAHC-modified GNP solution, and mixed solution of *E. coli* and HAHC-modified GNPs. Before mixing GNPs with *E. coli*, the absorbance spectrum of the HAHC-modified GNP solution exhibits a single surface plasmon resonance (SPR) band at 522 nm. After mixing, this SPR band is slightly redshifted (Δ*λ* = 4 nm), and another broad SPR band is observed in the near-infrared (NIR) region, indicating that the surface plasmons of GNPs couple with each other. The decrease in the interparticle distance between GNPs when GNPs are self-assembled on the bacteria surface after mixing is mainly responsible for this observation. To examine the driving force for the self-assembly of GNPs, zeta-potentials and transmission electron microscope (TEM) measurements were carried out (Fig. 2c and Figure S1). The zeta-potentials of *E. coli*, HAHC-modified GNPs, and citrate-capped GNPs were found to be − 44.2 ± 10.3, − 10.9 ± 7.8, and − 30.3 ± 2.2 mV, respectively. TEM images show that, unlike HAHC-modified GNPs, citrate-capped GNPs are not self-assembled on the surface of the bacteria. UV-vis spectra were also recorded before and after mixing citrate-capped GNPs with *E. coli* (Figure S2). The SPR band of citrate-capped GNPs did not shift, and no additional SPR band was observed in the NIR region. From these results, it can be concluded that citrate-capped GNPs did not assemble on the surface of *E. coli* due to strong electrostatic repulsion. It is generally considered that nanoparticles are approximately neutral when their zeta-potentials are less than ± 10 mV^23^. Therefore, the electrostatic repulsion between HAHC-modified GNPs and bacteria would be negligible. Therefore, hydrogen bonding between the hydroxyl group of hydroxylamine and the amine group of surface proteins or lipopolysaccharide^24,25^ would be responsible for the self-assembly of HAHC-modified GNPs on the *E. coli* surface. Note that HAHC-modified GNPs also self-assemble to form dense arrays on the surface of *Pseudomonas aeruginosa* (*P. aeruginosa*).

The nanoporous mirror for hydrodynamic cell trapping and signal amplifications was fabricated through successive depositions of metal and dielectric layers (Fig. 2d). First, an 80-nm-thick gold thin film was deposited on a porous polycarbonate (PC) membrane with a pore diameter of 400 nm by electron beam evaporation. Subsequently, different thicknesses (0.5, 1, 2, and 5 nm) of silicon dioxide (SiO_2_) layers, which act as spacers between the gold thin film and plasmonic bacteria, were deposited over the gold thin film by radio frequency (RF) sputtering. The deposition of the gold thin film and SiO_2_ layers was confirmed by reflectance (Figure S3a). The gold thin film exhibited high reflectance of infrared or near-infrared wavelengths. Moreover, evaluating the reflectance spectra with respect to SiO_2_ thickness shows that the reflectance intensity for the gold film is inversely proportional to the SiO_2_ thickness. This proportionality is due to the SiO_2_ layer acting as an antireflective layer and decreasing the reflectivity with increasing thickness of the SiO_2_ layer^26^. In addition, energy-dispersive X-ray spectroscopy (EDS) was carried out (Figure S3b). EDS elemental maps indicate that with the increase in SiO_2_ thickness, the Si atoms become more abundant on the surface of the membrane. The left image of Fig. 2e shows a representative SEM image of the resulting nanoporous mirror. Comparison with the SEM image of the bare porous PC membrane (before deposition) indicates that the pore size is slightly decreased by 3.7 nm (from 405.5 ± 22.9 nm to 401.8 ± 22.9 nm, Figure S4). This result confirms that a structural change such as pore blocking does not occur during the deposition. A representative SEM image of the plasmonic bacteria on a nanoporous mirror after filtering a solution of plasmonic bacteria through the nanoporous mirror is shown in the right image of Fig. 2e (a low-magnification image is also shown in Figure S5).

**Fig. 3:**
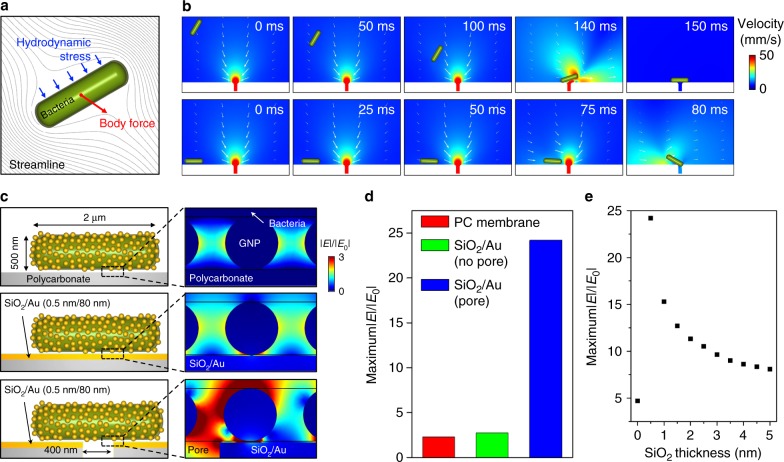
Fluid dynamics simulation of the hydrodynamic trapping of bacteria and electromagnetic field simulation of GNP-assembled bacteria on the nanoporous mirror **a** Schematic illustration of body force resulting from hydrodynamic stress, acting on bacteria in a flow. **b** Fluid dynamics simulation results when the centers of bacteria are 5 μm (top row) and 1 μm (bottom row) above the membrane. **c** Schematic illustration of plasmonic bacteria on (top) the bare PC membrane, (middle) SiO_2_/Au-coated PC membrane, and (bottom) SiO_2_/Au-coated porous PC membrane with corresponding EM field distribution images. **d** Normalized maximum electric field amplitude (|*E*|/|*E*_*0*_|) with respect to the condition of the membrane. **e** Calculated maximum |*E*|/|*E*_*0*_| with respect to SiO_2_ thickness

A two-dimensional fluid dynamics simulation was carried out for the hydrodynamic trapping of bacteria on the nanopores (Fig. 3a). Bacteria are assumed to be a rod with a diameter of 500 nm and a length of 2 μm. To observe the effect of the pore on the hydrodynamic trapping, the effect of fluid flow resulting from numerous pores and the wall of the fluidic channel is assumed to be negligible, and a single pore with a diameter of 400 nm in a large fluidic channel (diameter of 100 μm) is considered. The fluid flow and the concomitant movement of bacteria are calculated simultaneously by using a fluid structure interaction (FSI) method^27^. In this method, the incompressible Navier–Stokes equation is solved to calculate the fluid flow. At the same time, hydrodynamic stress on bacteria (Γ) is calculated to predict the movement of bacteria in a flow using the following equation,

1 $${\mathrm{\Gamma }} = \left[ { - pI + \mu (\nabla u_{{\mathrm{fluid}}} + (u_{{\mathrm{fluid}}})^T)} \right]$$

where *u*_*fluid*_, *p*, and *μ* are the fluid velocity, pressure, and kinematic viscosity, respectively. Finally, the body force (*F*) resulting in the movement of bacteria is calculated by the following equation,

2 $$F = \mathop {\int }\limits_V \nabla \cdot \sigma dV$$

where *dV* is an infinitesimal volume element and *σ* is a stress field. The stress at the surface of bacteria satisfies the boundary condition $\sigma \cdot n = {\mathrm{\Gamma }} \cdot n$, where *n* is a normal vector to the surface of bacteria.

Time sequence images of bacteria when the centers of bacteria are initially 5 μm (top row) and 1 μm (bottom row) above the membrane surface are shown in Fig. 3b. In both cases, owing to the hydrodynamic pressure acting on the bacteria surface, bacteria are forced to move along the flow and finally trapped on the nanopore. Irrespective of the initial location of bacteria relative to the nanopore (i.e., different orientation and distance), bacteria are found to be trapped on the pore in the end (Figure S6, Supplementary Movie 1, and Supplementary Movie 2).

To estimate the optical amplifications of the plasmonic bacteria on a nanoporous mirror, an electromagnetic (EM) simulation was conducted. Figure 3c shows the simulation schemes for plasmonic bacteria on different membranes (PC membrane without nanopores, SiO_2_ and gold thin films (SiO_2_/Au) on the PC membrane without nanopores, and the nanoporous mirror) and their EM field distributions (the dotted square box in each scheme). The dimensions of bacteria are identical to those used for the fluid dynamics simulation. The diameter of the GNP on plasmonic bacteria is fixed at 20 nm. The thicknesses of SiO_2_ and gold films are 0.5 and 80 nm, respectively. The circular pore diameter is fixed at 400 nm in accordance with our experimental conditions. In the EM simulation, the local electric field enhancement (|*E*|/|*E*_*0*_|) from the ratio of the near field (|*E*|) and the incident field (|*E*_*0*_|) is calculated. The EM field distribution images indicate that when SiO_2_ and Au are deposited on the membrane, the local electric field enhancement increases, particularly in the space between SiO_2_/Au and the GNPs because of the strong coupling between the two surface plasmons (i.e., the GNPs and gold film). Interestingly, with the presence of the nanopores, the field enhancement significantly increases. As nanopores are known to act as a diffraction grating at the fringe of the pore^28–30^, constructive interference between incident light and its diffraction can induce field enhancement. This possibility is also supported by the field enhancement observed in the low-magnification EM field distribution images in Figure S7.

The EM simulation results were quantitatively analyzed for the comparison of the maxima of the local electric field enhancement in the space between bacteria and the membrane for each case (Fig. 3d). The maximum local electric field enhancement values were found to be 2.33, 2.77, and 24.20, respectively. This result indicates that the presence of both the nanopore and SiO_2_/Au on the membrane increase the local field enhancement 10.4 times. On the other hand, owing to the contribution of the nanopore alone, the local field enhancement is estimated to be increased 8.7 times. These results suggest that in our design, the contribution of the constructive interference to the electric field amplification would be comparable to that of the plasmon coupling between the GNPs and the gold film. According to our fluid dynamics simulation, most bacteria are expected to be located on the nanopores during the filtration. Therefore, it is reasonable to assume that in our design, the optical signal of the plasmonic bacteria on a nanoporous mirror would originate mainly from the nanopore. An EM simulation of a similar design without GNP was also conducted (Figure S8). The effect of GNPs on the local electric field enhancement was maximized when the nanoporous mirror was used.

The near-field enhancement between the GNPs and the gold film is known to be affected by the SiO_2_ thickness^31^. To examine the effect of SiO_2_ thickness, an EM simulation was also carried out with different SiO_2_ film thicknesses (from 0 to 5.0 nm). Figure 3e shows the maxima of |*E*|/|*E*_*0*_| with respect to the SiO_2_ thickness. Figure S9 also shows the EM field distribution images. When decreases in the thickness of SiO_2_ from 5 nm to 0.5 nm, the maximum local electric field enhancement exponentially increases. This change is related to the distance-dependent weakening of the plasmon coupling between the GNPs and gold film. Note that the dramatic decrease in the near-field enhancement in the absence of the SiO_2_ film is related to the formation of a contact where electric conduction occurs. The electric conduction significantly weakens the strength of the plasmon coupling^32–34^. In addition, we have investigated how the maximum of electric field enhancement varies with changes in the wavelength of the incident light by EM simulation. As shown in Figure S10, the maxima of the electric field enhancement increased as the thickness of SiO_2_ decreased from 5 to 0.5 nm. No noticeable shift was observed, irrespective of the wavelength of the incident light.

**Fig. 4:**
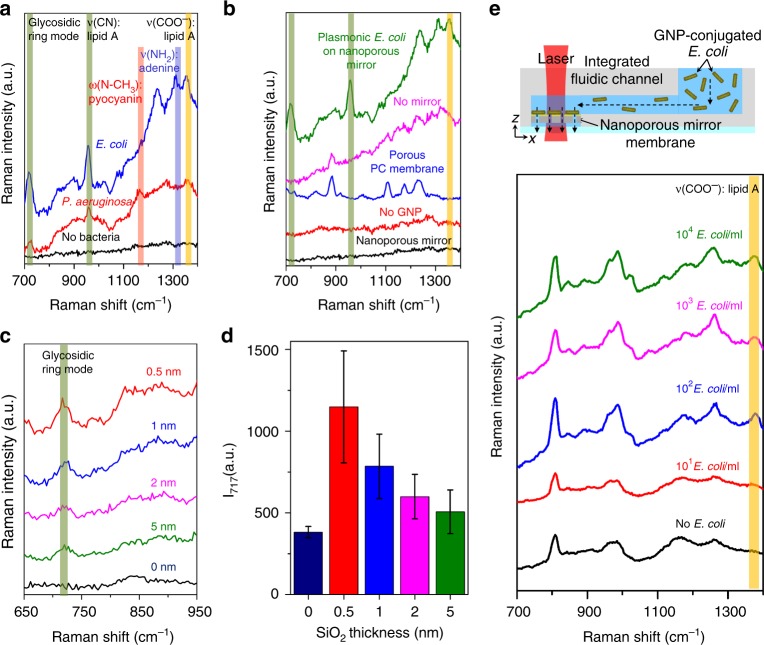
Raman measurement of plasmonic bacteria on nanoporous mirror **a** Raman spectra of nanoporous mirror with no bacteria (black), plasmonic *P. aeruginosa* on the nanoporous mirror (red), and plasmonic *E. coli* on the nanoporous mirror (blue). Green and yellow boxes indicate the common Raman transitions of *E. coli* and *P. aeruginosa*. Red and blue boxes indicate the distinctive Raman transitions of *P. aeruginosa* and *E. coli*, respectively. **b** Raman spectra of the nanoporous mirror with no bacteria (black), bare *E. coli* on the nanoporous mirror (no GNPs, red), the porous PC membrane (blue), plasmonic *E. coli* on the porous PC membrane (magenta) and plasmonic *E. coli* on the nanoporous mirror (green). **c** Raman spectra of plasmonic *E. coli* on the membrane with different SiO_2_ thicknesses (from the top, 0.5, 1, 2, 5, and 0 nm). **d** Raman intensity at 717 cm^−1^ with respect to SiO_2_ thickness. **e** Schematic illustration of the nanoporous mirror-integrated fluidic channel and Raman spectra measured after filtering 1 ml of plasmonic *E. coli* solution through the channel

To realize the benefits of plasmonic bacteria on a nanoporous mirror, this technique was applied to the detection of *E. coli* and *P. aeruginosa* via SERS. These bacteria were selected as they are involved in most waterborne diseases. First, plasmonic bacteria were quickly prepared and filtered through the nanoporous mirror. Then, Raman spectra were obtained from the membrane surface. Fig. 4a shows the representative Raman spectra of two bacteria on the surface of the membrane. Three Raman transitions for both bacteria are commonly observed at 717 cm^−1^, 958 cm^−1^, and 1351 cm^−1^, which correspond to a glycosidic ring mode, *ν*(CN), and *ν*(COO^−^), respectively^17–20^. Note that the structure of the cell membrane that is responsible for the observed SERS signals is similar for both bacteria as they are gram-negative^35^. Distinctive Raman transitions of *E. coli* and *P. aeruginosa* are also found at 1312 cm^−1^ and 1155 cm^−1^, respectively. These Raman transitions can be assigned to *ν*(NH_2_) of adenine^20^ for *E. coli* and *ω*(N-CH_3_) of pyocyanin^36^ for *P. aeruginosa*.

To further elucidate the effects of the GNPs, mirror, and nanopores on the observed SERS enhancement, bare *E. coli* (*E. coli* without GNPs) and GNP-assembled *E. coli* (i.e., plasmonic *E. coli*) were filtered through the nanoporous mirror and porous PC membrane, respectively. Raman spectra were measured. As shown in Fig. 4b, Raman transitions that are apparent for the plasmonic *E. coli* on the nanoporous mirror are not observed in either case (i.e., bare *E. coli* on the nanoporous mirror and plasmonic *E. coli* on the porous PC membrane). These results strongly imply that the nanoporous mirror or GNPs do not induce strong SERS enhancement but, instead, the synergistic contributions of the plasmonic bacteria on a nanoporous mirror is mainly responsible for the observed SERS enhancement.

To compare the signal enhancement with respect to SiO_2_ thickness with the simulation result, a solution of GNP-assembled *E. coli* was filtered by the nanoporous mirrors with different SiO_2_ thicknesses, and Raman spectra were obtained (Fig. 4c). Similar to the EM simulation result, the intensity at 717 cm^−1^ increased as the SiO_2_ thickness decreased (Fig. 4d). No Raman transition peak was observed from the membrane without a SiO_2_ layer.

To investigate the sensitivity and detection time of our design, the plasmonic bacteria on a nanoporous mirror were further integrated into a simple fluidic channel. The fact that most bacteria are enriched on the nanoporous mirror was also confirmed by culturing the suspensions of bacteria before and after filtration (Figure S11). After the conjugation of GNPs onto *E. coli*, 1 ml of plasmonic *E. coli* solution was passed via the integrated fluidic channel, and Raman signal was directly measured. Figure 4e shows the Raman spectra at different concentrations of *E. coli*. All Raman spectra were analyzed on the basis of the Raman transition of *ν*(COO^−^) because the other Raman peaks show either weak intensity or strong interference with the Raman peaks of the fluidic channel materials (e.g., poly(methyl methacrylate), PMMA). From the Raman transition of *ν*(COO^−^), the limit of detection is found to be 10^2^ cells/ml. To examine the reproducibility of the SERS signal, SERS signals at four random spots were measured after filtration (spot size of laser: 0.2 mm, Figure S12a). Then, the average and standard deviation of the normalized Raman intensity of PMMA (987 cm^−1^, C–C stretching) were calculated (Figure S12b). The average and standard deviation calculated after filtering 10^2^ ~ 10^4^ *E. coli*/ml are 0.85 ± 0.02, 0.81 ± 0.08, and 0.92 ± 0.04, respectively. The standard deviation values indicate that the SERS signals from the plasmonic bacteria on a nanoporous membrane are quite reproducible. Note that a PC membrane with a smaller size (4 mm in diameter in our experiment) would slightly improve the reliability and reproducibility of the SERS signals. To evaluate the quantification of our method, the Raman transition of bacteria at 1351 cm^−1^, which can be assigned to *ν*(COO^−^) of lipid A, was normalized to 4 different Raman transitions of PMMA as internal standards (600 cm^−^^1^, 812 cm^−^^1^, 964 cm^−1^, and 1448 cm^−1^, corresponding to *ν*(O–C═O), *ν*(C–C–C–C), *ν*(C–C), and *ν*(CH_2_), respectively)^37^. As shown in Figure S13, the normalized Raman intensities generally increase with increasing concentration of bacteria, regardless of which Raman peak of PMMA is used as an internal standard. Regarding the detection time of our integrated platform, each step, that is, the conjugation between the GNPs and bacteria (i.e., the formation of plasmonic bacteria), the enrichment, and the detection, takes ~ 9 min, 30 s, and 5 s, respectively. The total detection time is shorter than *ca*. 10 min. Note that this detection time could be shortened by improving the conjugation time.

## Discussion

In conclusion, we have designed an integrated platform of a plasmonic bacteria on nanoporous mirror membrane via hydrodynamic trapping for the enrichment of bacteria and strong signal amplifications. HAHC-modified GNPs spontaneously self-assembled to form dense arrays on the surface of bacteria, such as *E. coli* and *P. aeruginosa*, in order to amplify the optical signal. Fluid dynamics simulations reveal that bacteria are trapped on the nanopore during filtration, resulting in the enrichment. EM simulations show that, owing to the nanopore geometry, the electric field is enhanced > 10 times compared with that without the nanopores. The near-field amplifications of our design can be attributed to (1) the focused light by constructive interference between incident light and its diffracted one via the nanopore geometry, (2) self-assembled nanoplasmonic optical antennas on the surface of the bacteria, and (3) the plasmonic mirror. Our plasmonic bacteria on a nanoporous mirror platform was successfully applied to the detection of *E. coli* and *P. aeruginosa*. Through further integration into a fluidic channel, trace *E. coli* concentrations as low as 10^2^ cells/ml are detectable via SERS. We believe that our integrated optofluidic platform paves the way for the rapid, precise identification of various pathogens and further understanding of microbial resistance for effective treatment as the platform can provide insights regarding microbial surface expression to correlate with the genome of emerging pathogens and the effects of the water surface and the environmental impacts of the transmission line.

## Materials and methods

### Synthesis of HAHC-modified GNPs

GNPs modified with HAHC were synthesized based on the previously reported method^38^. In brief, for the synthesis of seed particles, 125 ml of 254 μM HAuCl_4_∙3H_2_O solution was prepared with deionized (DI) water and boiled in a 250 ml round-bottom flask in an oil bath at reflux with vigorous stirring. In addition, 12.5 ml of 40 mM trisodium citrate dehydrate prepared with DI water was rapidly added to the flask with continuous vigorous stirring for 10 min, and the solution was stirred for 15 min at room temperature (RT). For the growth and replacement of citrate with HAHC, 30 ml of seed particle solution and 3 ml of 0.2 M HAHC were subsequently added to 270 ml of DI water with vigorous stirring at RT. Then, 2.5 ml of 25.4 mM HAuCl_4_∙3H_2_O solution was added dropwise to the mixture for 1 min.

### Fabrication of the nanoporous mirror

The nanoporous mirror was fabricated through two steps of serial deposition of a gold (Au) thin film and a SiO_2_ layer. First, the PC porous membrane (Cyclopore 400-nm pore size, GE Healthcare Bio-Sciences, PA, USA) was loaded in an electron beam evaporation for the gold thin film deposition. A 5-nm-thick titanium layer, as an adhesion layer, and an 80-nm-thick Au film were sequentially deposited on the PC porous membrane under a base pressure of 2 × 10^−7^ Torr. Afterwards, SiO_2_ layers with different thicknesses (0.5, 1, 2, and 5 nm) were deposited over the thin Au film by RF sputtering. The reflectance spectra of the nanoporous mirrors were measured using a UV-VIS-NIR scanning spectrophotometer (UV-3101PC, Shimadzu, MD, USA), and the EDS was carried out for the elemental mapping of Si atoms using an EDS attached to the SEM/FIB (Quanta 3D FEG, OR, USA).

### Fluid dynamics simulation

For the examination of the hydrodynamic trapping of bacteria on nanopore, the fluid flow and the movement of bacteria were calculated simultaneously by using a FSI method^27^. Bacteria was assumed to have a rod shape with a diameter of 500 nm and a length of 2 μm. The membrane was assumed to have a single pore with a diameter of 400 nm in a chamber with a diameter of 100 μm.

### EM simulation

To investigate the optical properties of GNP-assembled bacteria (plasmonic bacteria) on the nanoporous mirror, wave optics simulations were carried out using the commercial EM simulation package (COMSOL Multiphysics). The complex values of the wavelength-dependent refractive index of bulk gold were taken from the database reported by Johnson and Christy^39^. The refractive index values of SiO_2_ and PC were set to be 1.45 and 1.56, respectively. The *p*-polarized EM light was incident to the surface of the nanoporous membrane. The wavelength of incident light was set to be 785 nm. In this simulation, we assumed that the GNPs attached on the surface of bacteria were uniformly distributed with an interparticle distance of 10 nm.

### Bacterial growth

*E.* *coli* and *P. aeruginosa* were grown in Lysogeny broth medium at 37°C with shaking (250 ml Erlenmeyer flasks, 150 rpm). When the culture turbidity (OD 600) reached 1.0, bacterial cells were harvested by centrifugation (4500 rpm, 10 min), and *E. coli* and *P. aeruginosa* cell pellets were suspended in DI water, followed by three washes with DI water for evaluating the SERS application.

### Design and fabrication of integrated fluidic channel

The integrated fluidic channel was designed as shown in Figure S14. The diameter and the height of the detection zone were 4 mm and 0.28 mm, respectively. To integrate the channel into a disposable cartridge, PMMA sheets and PC sheets were used for the top and bottom layers, respectively. Top and bottom layers were cut with a VersaLASER VL-200 laser cutting system (Universal Laser System, Inc., Scottsdale, AZ, USA). All samples were cleaned with 70% ethanol twice for 10 min, rinsed with DI water, and dried using N_2_. Then, the top and bottom layers were bonded together using 80 μm-thick double-sided tape (ARcare® 90445, Adhesives Research, Inc., Glen Rock, PA, USA). To load the sample using a syringe, a loading zone was made of polydimethylsiloxane (PDMS) and introduced at the top layer of the fluidic channel. Finally, the nanoporous mirror was bonded to the bottom region of the fluidic channel with the double-side tape.

### Raman measurement

For Raman measurement, a commercial Raman spectrometer (QE65000 from Ocean Optics Inc.) and a 785 nm laser module (I0785MM0350MS from Innovative Photonic Solution Inc.) were used. A 785 nm laser operated at a power of 250 mW was used in the Raman measurements with 5 s of integration time.

## Electronic supplementary material

Supplementary Information

Supplementary Movie 1

Supplementary Movie 2
