# Supplementary material for: Plasmonic bacteria on a nanoporous mirror via hydrodynamic trapping for rapid identification of waterborne pathogens
Source: Light Sci Appl. 2018 Oct 3;7:68. doi: 10.1038/s41377-018-0071-4 (PMC6168555; doi:10.1038/s41377-018-0071-4)
Supplement: Supplementary file 1 — Supplementary Information [file 41377_2018_71_MOESM1_ESM.docx]

**Supplementary Information:**

**Plasmonic Bacteria on a Nanoporous Mirror *via* Hydrodynamic Trapping for Rapid Identification of Waterborne Pathogens**

*Keumrai Whang^1ǂ^, Jong-Hwan Lee^2ǂ^, Yonghee Shin^1ǂ^, Wooju Lee^3^, Young Wan Kim^3^, Dongchoul Kim^3*^, Luke P. Lee^2*^, Taewook Kang^1*^*

^1^Department of Chemical and Biomolecular Engineering, Sogang University, Seoul 04107, Korea.

^2^Biomolecular Nanotechnology Center, Berkeley Sensor and Actuator Center, Department of Bioengineering, University of California, Berkeley, Berkeley, CA 94720, USA.

^3^Department of Mechanical Engineering, Sogang University, Seoul 04107, Korea

*E-mail: dckim@sogang.ac.kr

*E-mail: lplee@berkeley.edu

*E-mail: twkang@sogang.ac.kr


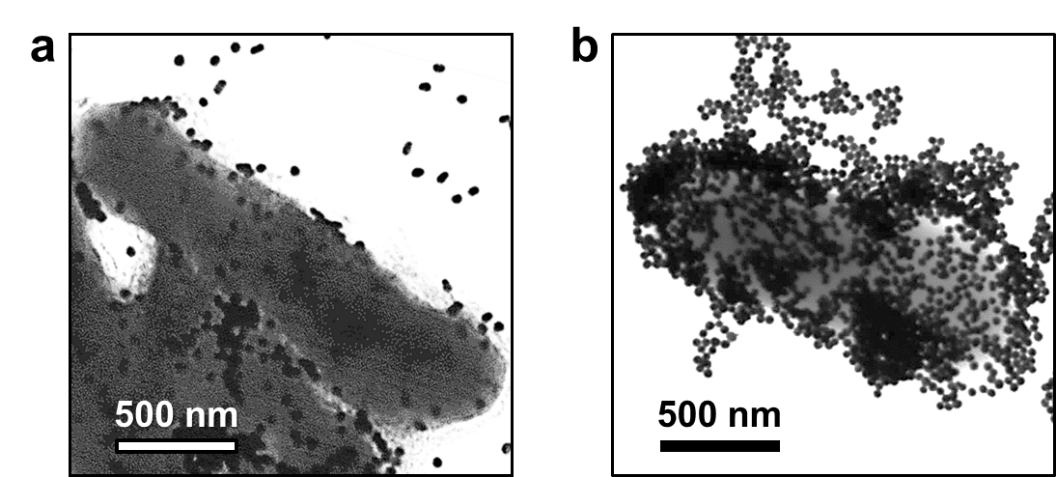


**Figure S1.** TEM images taken after mixing *E. coli* with (a) citrate-capped GNP solution or (b) HAHC-modified GNP solution.


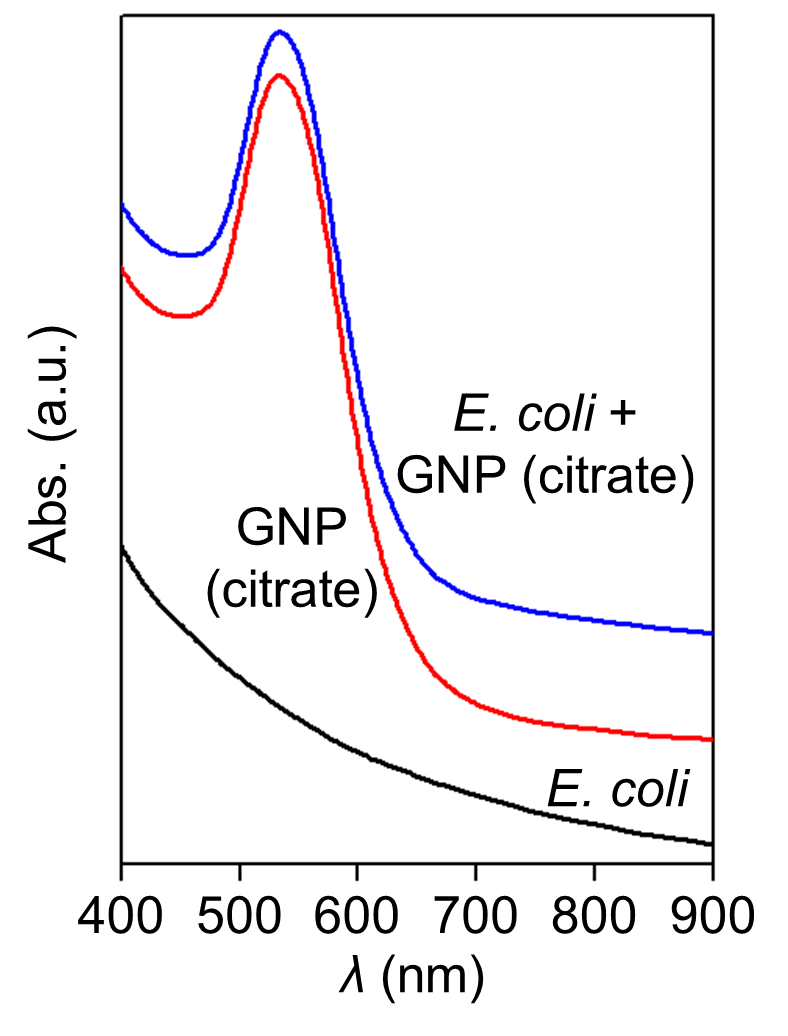


**Figure S2. UV-vis spectra taken before and after mixing citrate-capped GNP with *E. coli*.** UV-vis spectra of *E. coli* suspension (black), citrate-capped GNP solution (red), and mixed solution of *E. coli* suspension and citrate-capped GNP solution (blue).

**
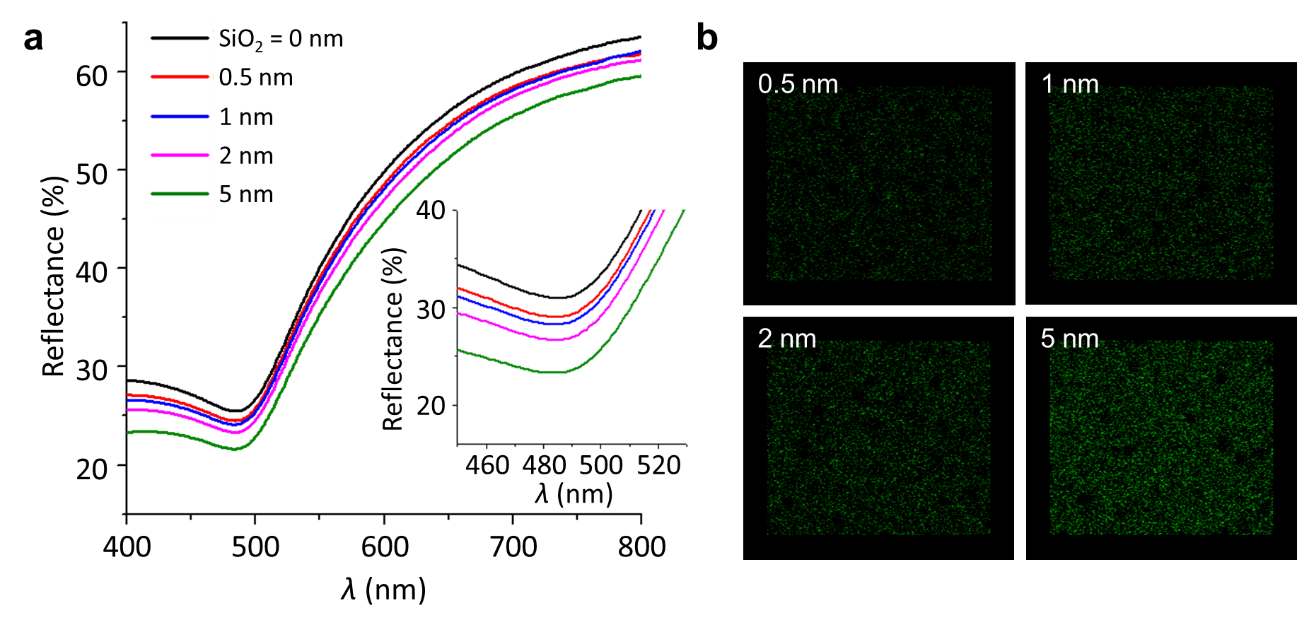
**

**Figure S3. Characterization of porous PC membranes after deposition of gold film with different thickness of SiO_2_ thin film.** (a) Reflectance spectra of porous PC membrane after deposition of gold film and different thicknesses of SiO_2_ thin film (0, 0.5, 1, 2, and 5 nm). (b) Atomic-scale elemental maps of Si atom on the porous PC membrane after deposition of gold film and different thicknesses of SiO_2_ thin film (0.5, 1, 2, and 5 nm) using EDS.

**
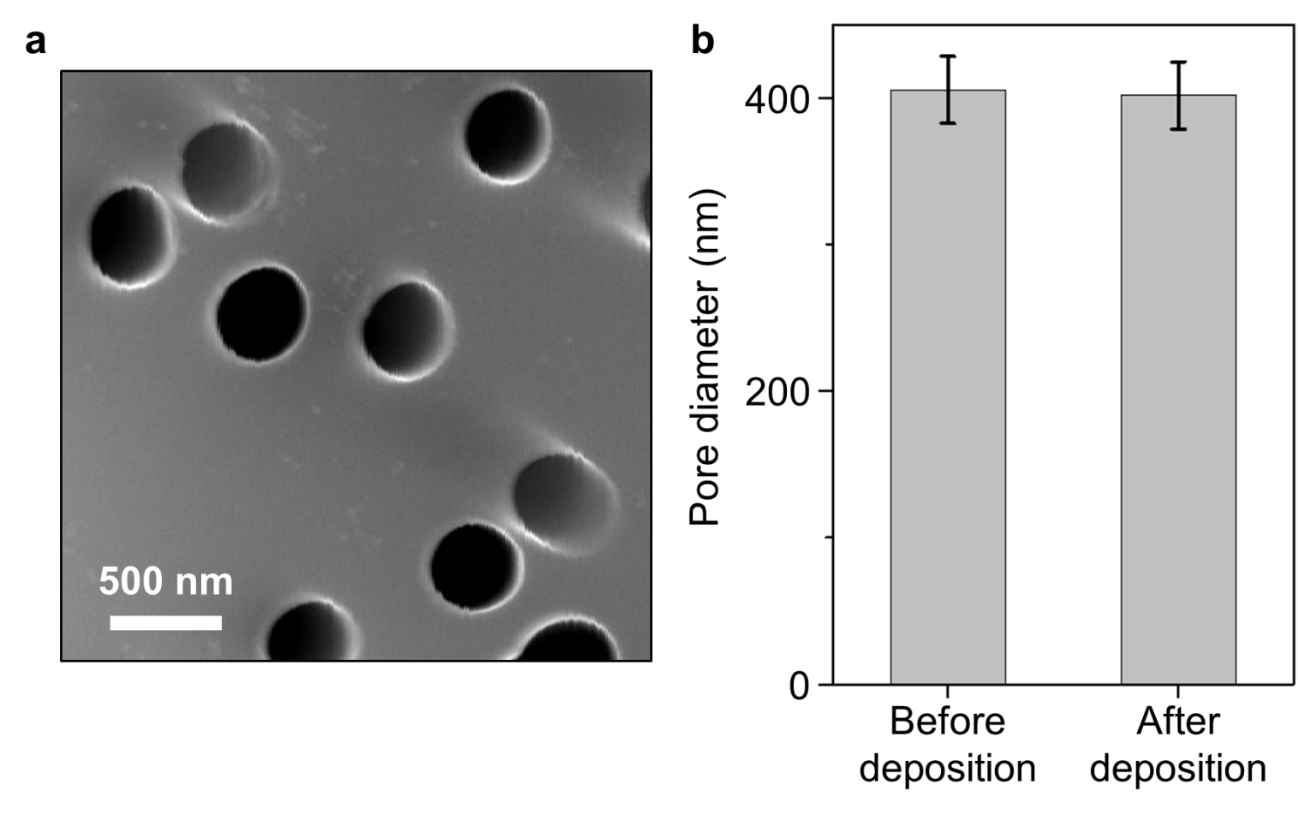
**

**Figure S4. Characterization of porous PC membrane before and after deposition of gold and SiO_2_ film.** (a) Representative scanning electron microscope image before deposition. (b) Average diameters of the pores (PC membrane) before and after deposition of gold and SiO_2_ thin film (20 pores are considered for each membrane).


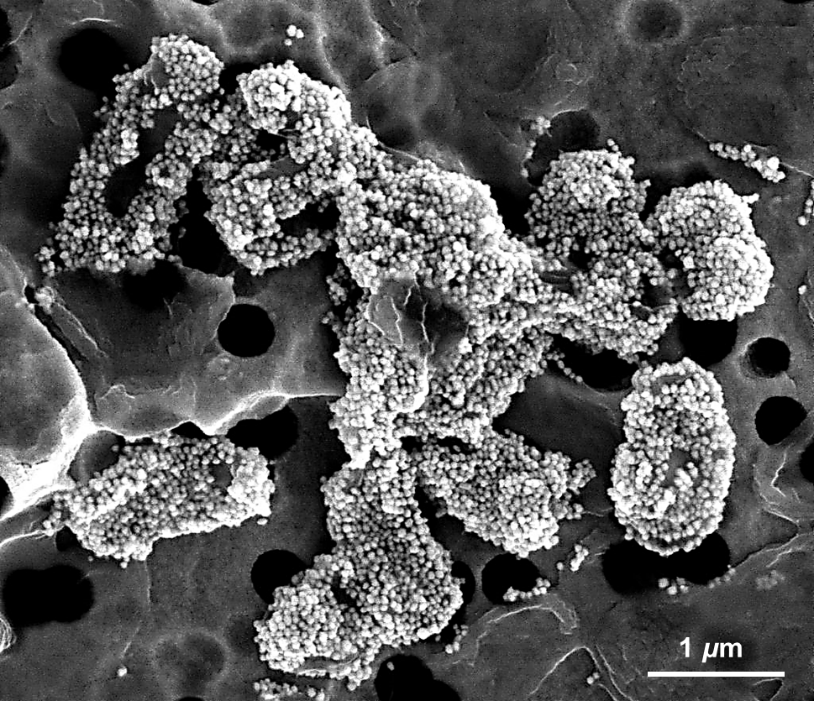


**Figure S5.** Representative scanning electron microscope image of GNP-assembled *E. coli* at low magnification. The GNP-assembled *E. coli* were enriched on the nanoporous mirror *via* hydrodynamic trapping.

**
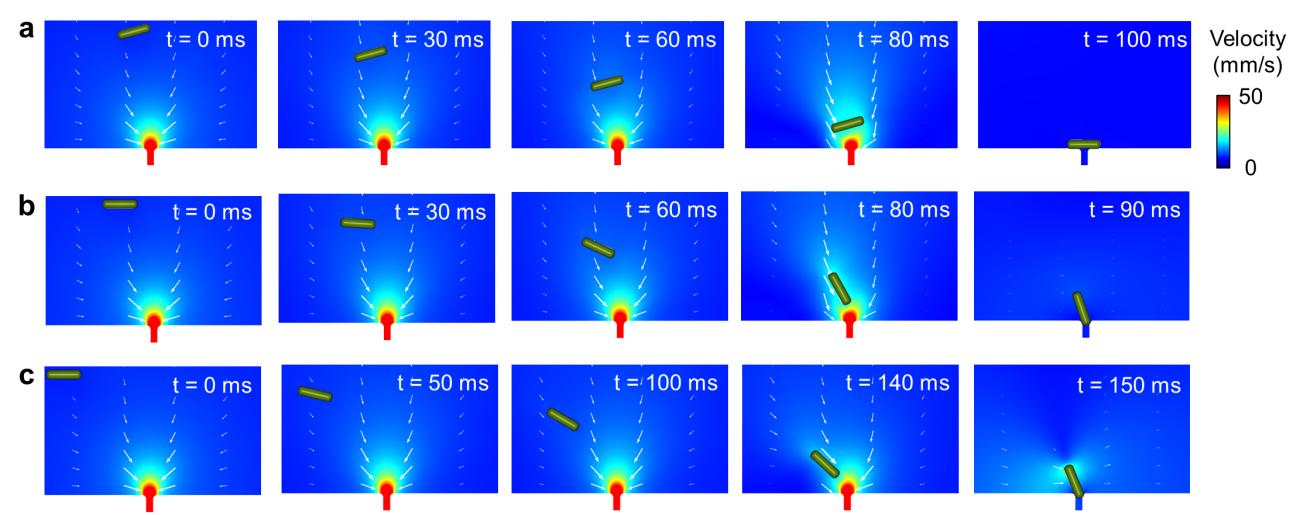
**

**Figure S6. Fluid dynamics simulation for bacteria at different initial positions.** Fluid dynamics simulation results at (a) 1 *μ*m from the center of the pore in x-direction with the bacteria tilted initially, (b) 1 *μ*m and (c) 7 *μ*m from the center of the pore in x-direction with the bacteria not tilted initially.

**
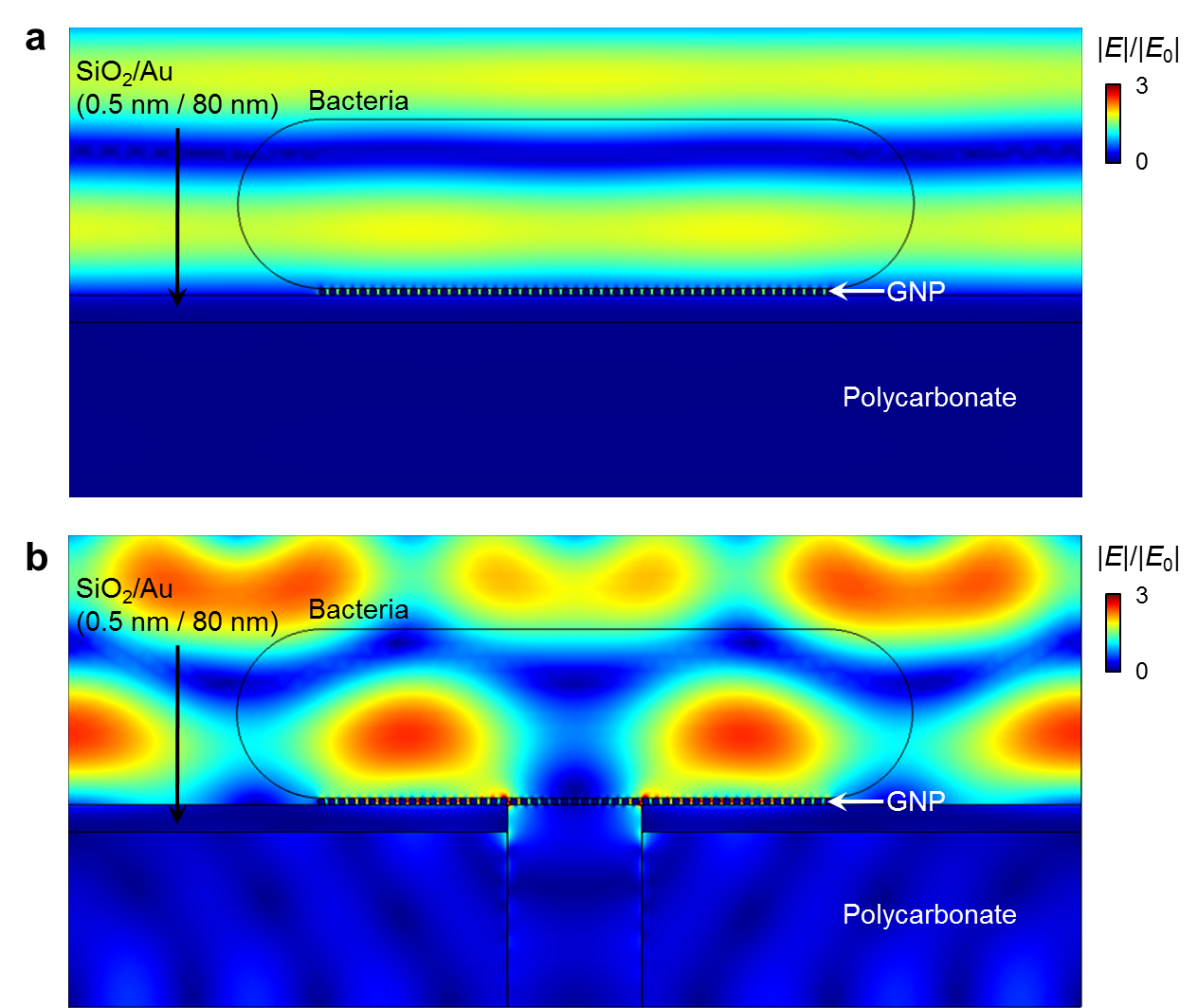
**

**Figure S7. Electromagnetic (EM) simulation (low magnification).** EM simulation of plasmonic bacteria on polycarbonate membrane (a) without pore and (b) with a pore.


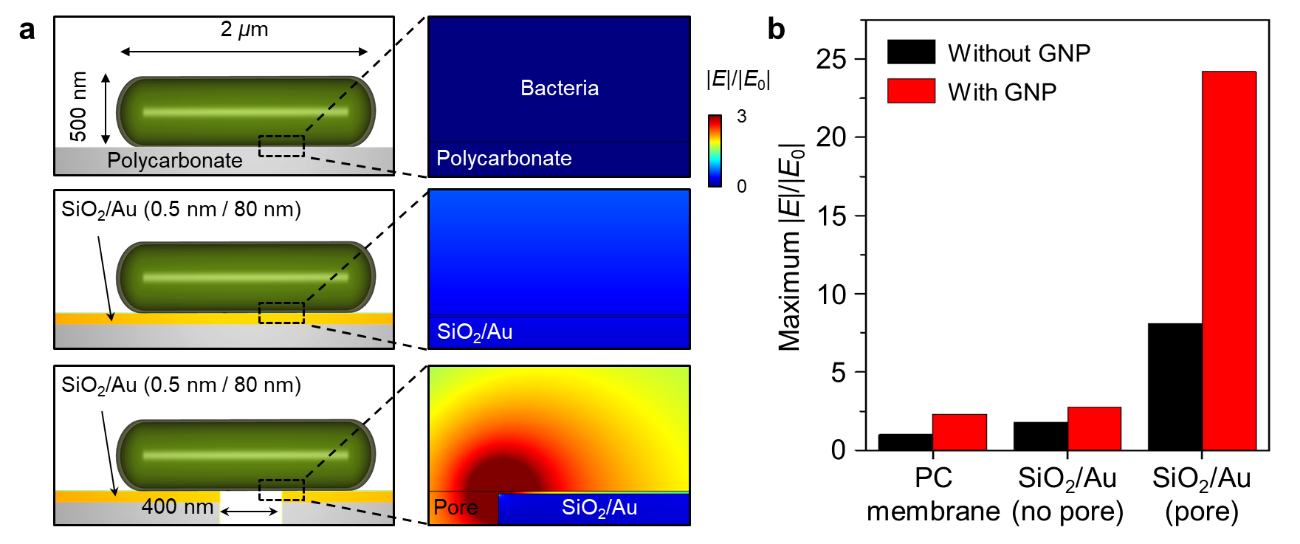


**Figure S8.** (a) Schematic illustration of bare bacteria on (top) bare PC membrane, (middle) SiO_2_/Au coated PC membrane, and (bottom) SiO_2_/Au coated porous PC membrane with corresponding EM field distribution images. (b) Normalized maximum electric field amplitude (|*E*|/|*E_0_*|) with respect to the condition of membrane.

**
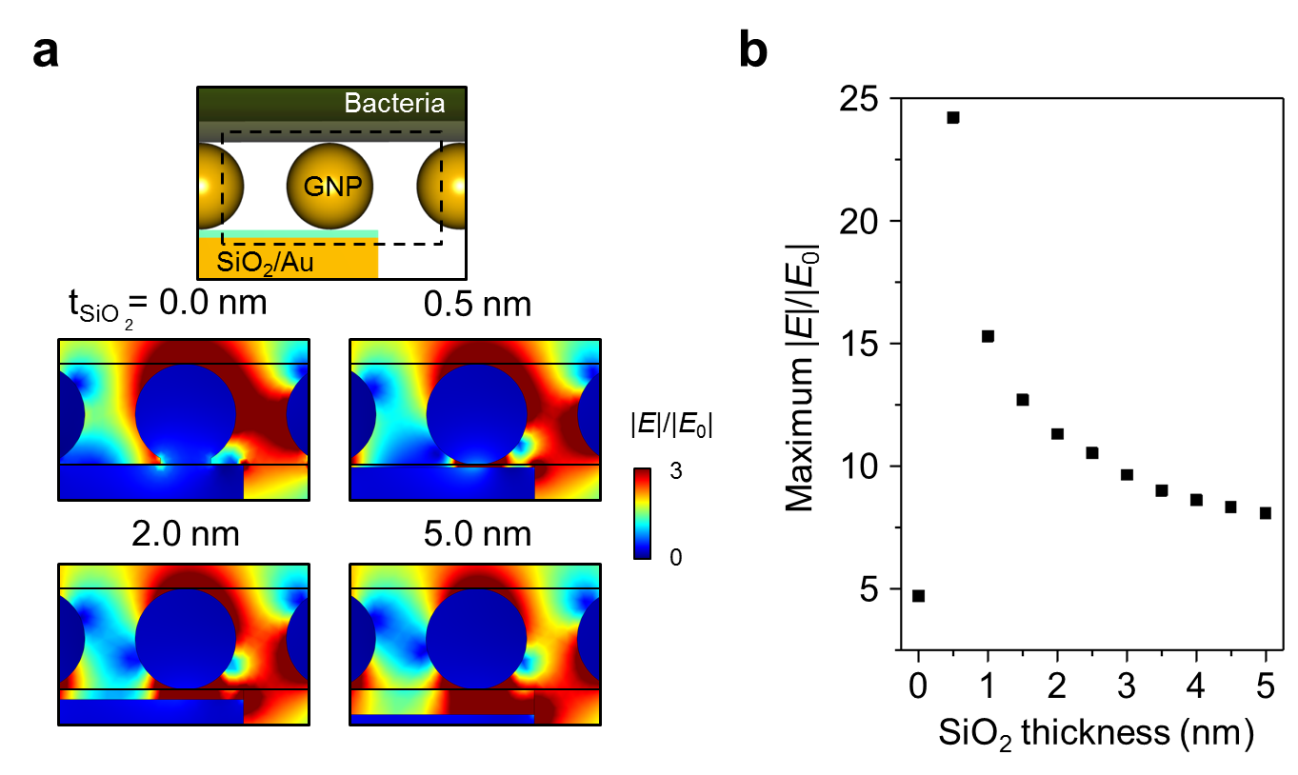
**

**Figure S9. Electromagnetic (EM) simulation results with different thickness of SiO_2_ film.** (a) Schematic illustration showing the area where the EM simulation was conducted and corresponding EM field distribution images with SiO_2_ thicknesses of 0.0, 0.5, 2.0, and 5.0. (b) Calculated maximum |*E*|/|*E_0_*| with respect to SiO_2_ thickness.


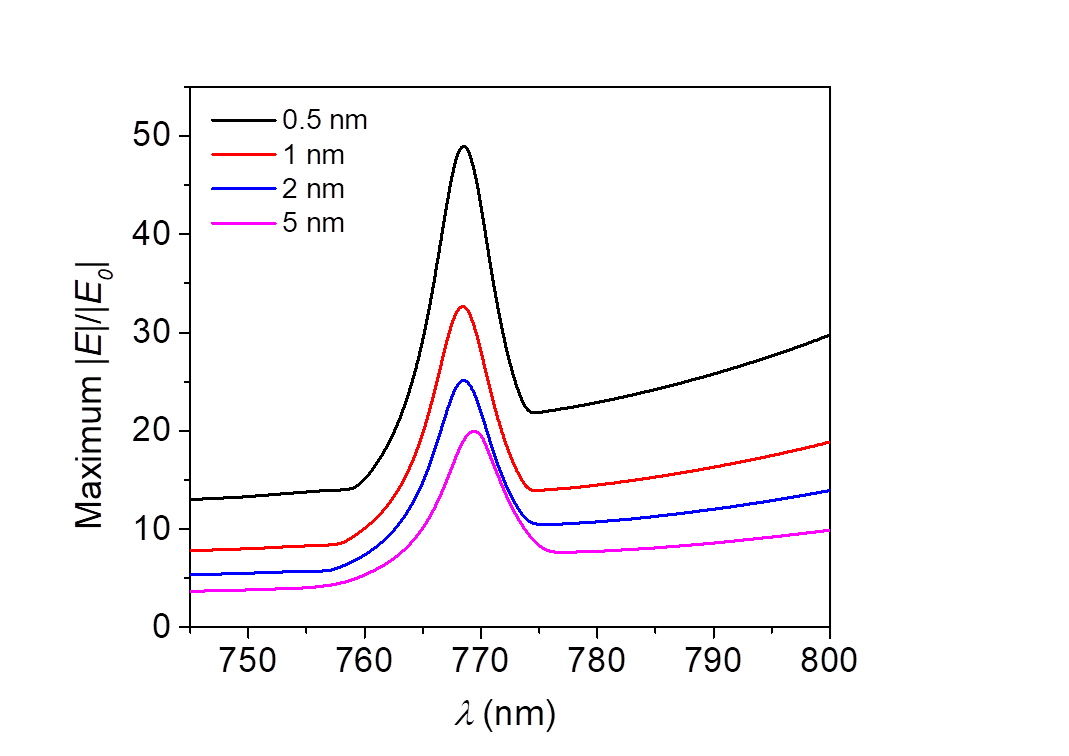


**Figure S10.** Maximum local electric field enhancement of plasmonic bacteria on a nanoporous mirror with respect to the wavelength of an incident light

**
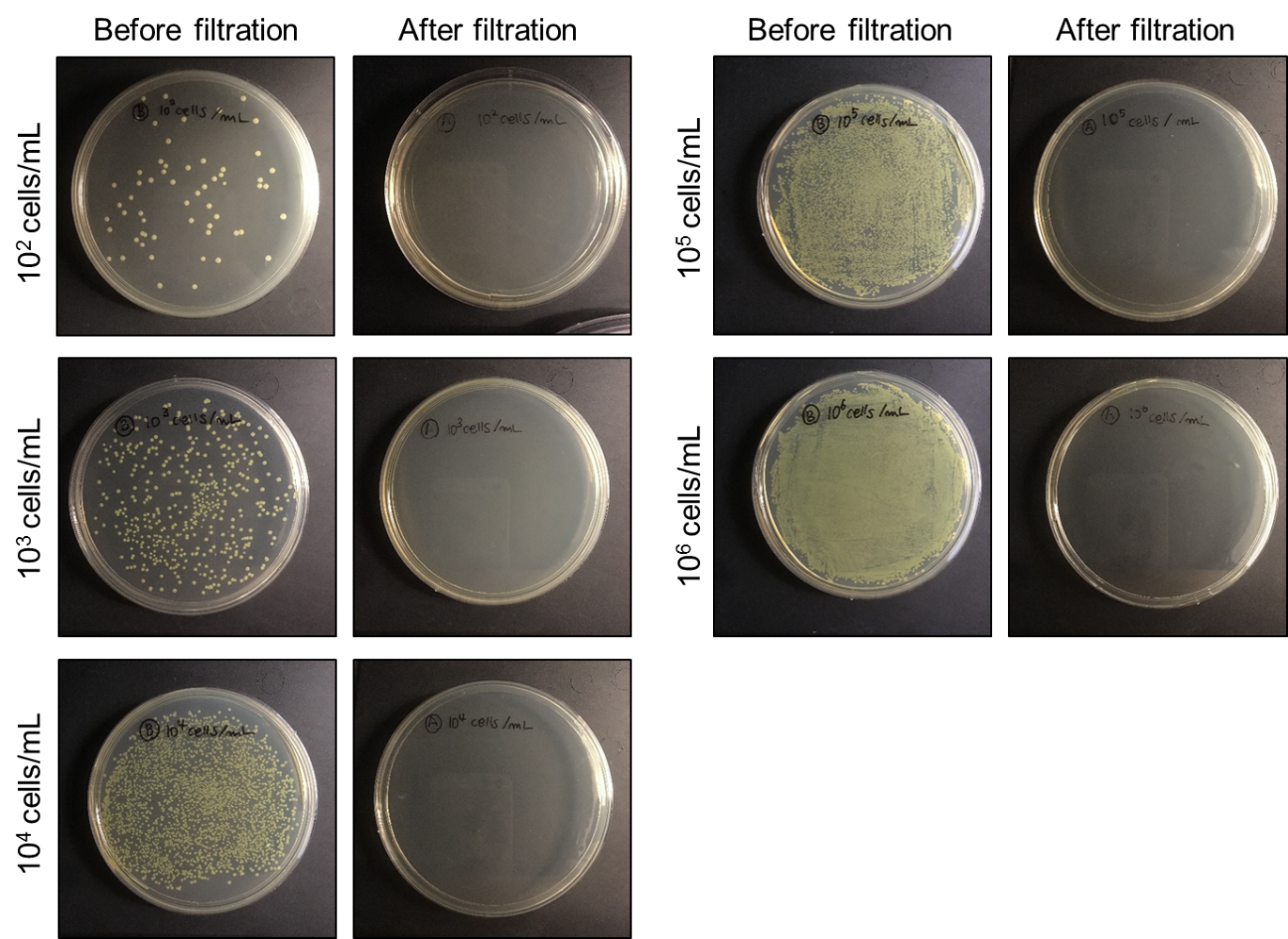
**

**Figure S11.** Bacterial (*E. coli*) colony growth on agar plate before and after filtering 1 ml of *E. coli* suspension with different concentrations through nanoporous mirror.

**
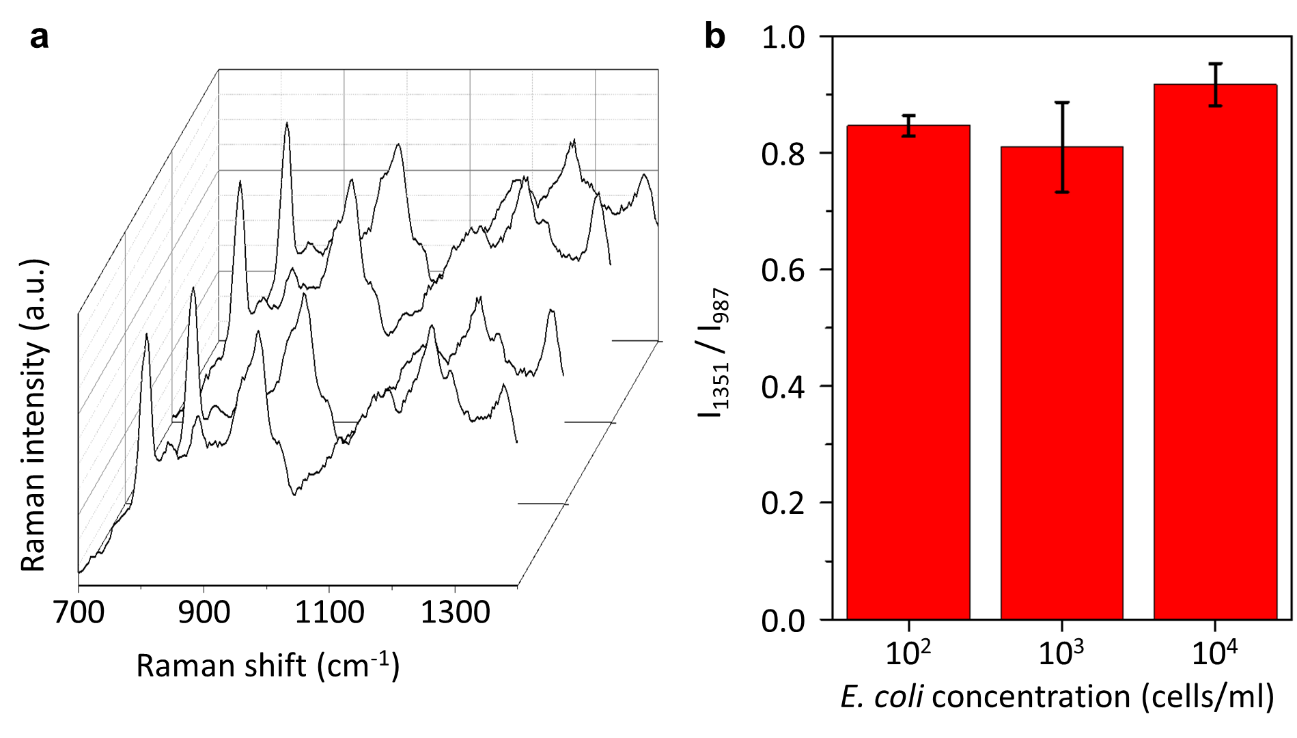
**

**Figure S12.** (a) Raman spectra obtained at 4 random spots after filtering 1 ml of 10^2^ *E. coli*/ml. (b) Average and standard deviation calculated from Raman signal of *ν*(COO^-^) (1351 cm^-1^) at 4 random spots after filtering 1 ml of 10^2^ ~ 10^4^ *E. coli*/ml normalized with Raman signal of polymethylmethacrylate (987 cm^-1^, C-C stretching).


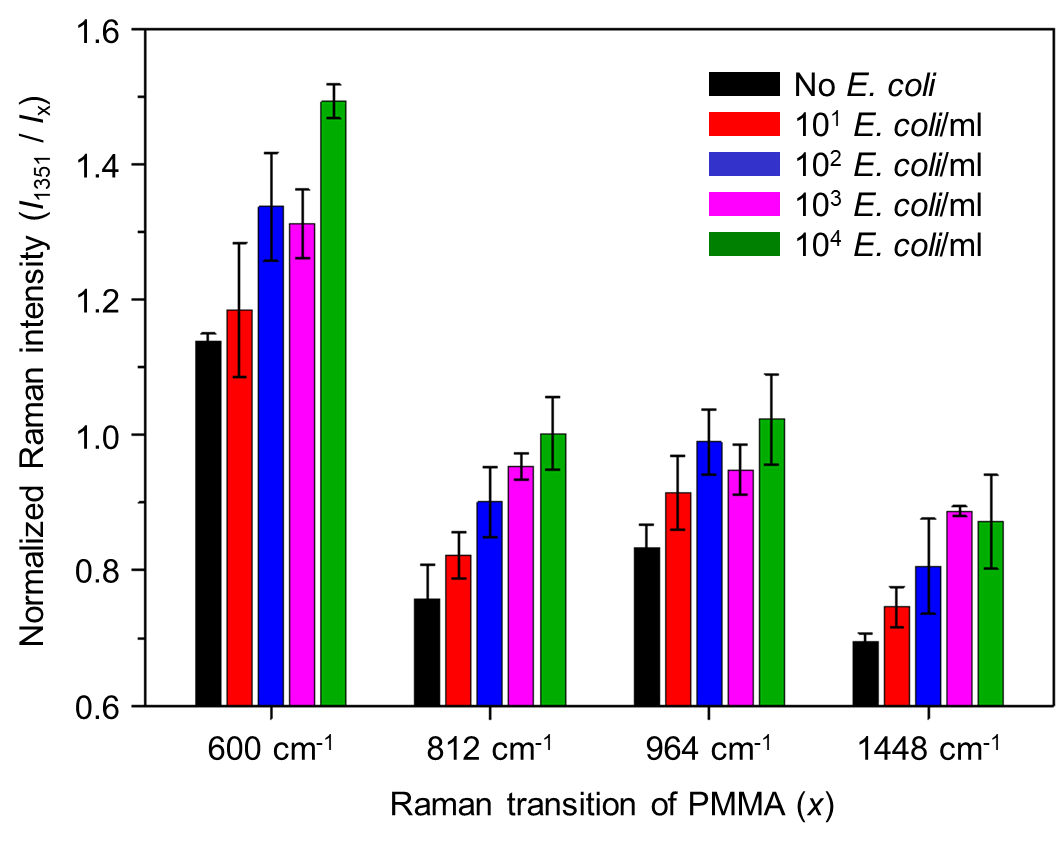


**Figure S13.** Normalized Raman intensity of different concentration of *E. coli* at 1351 cm^-1^ by using four different Raman transitions of PMMA at 600 cm^-1^, 812 cm^-1^, 964 cm^-1^, and 1448 cm^-1^ as internal standards, respectively.

**
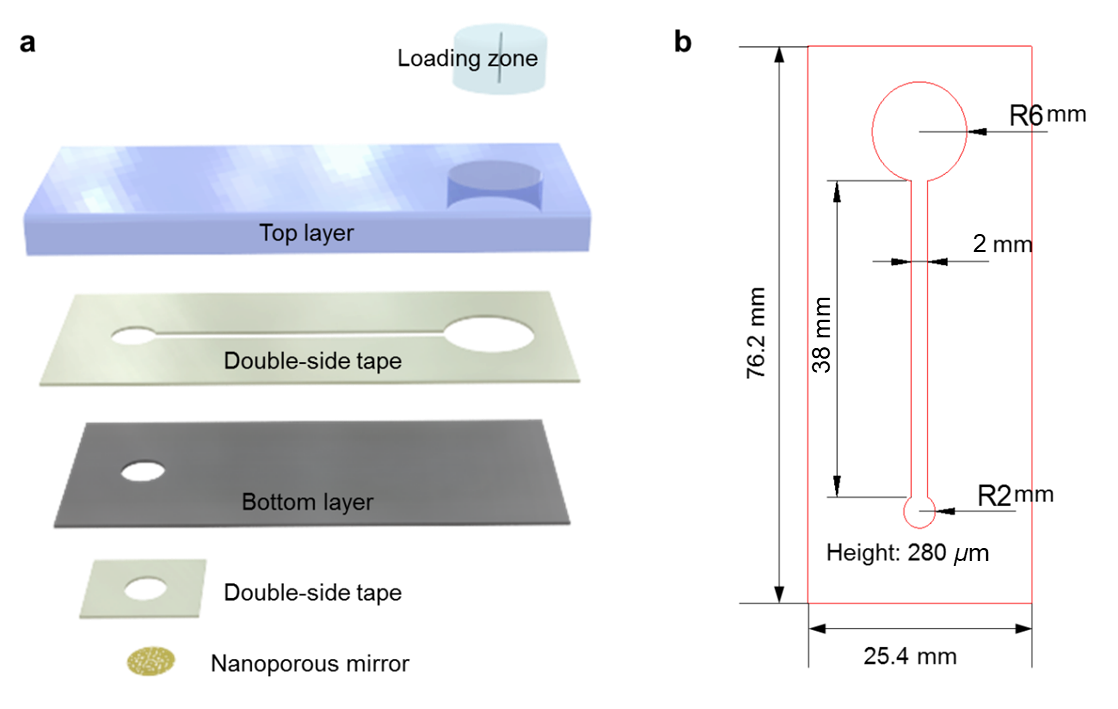
**

**Figure S14. Components and dimensions of the integrated fluidic channel.** (a) Schematic illustration of components of the fluidic channel. (b) Dimensions of the integrated fluidic channel.
